# Supplementary material for: MCM2-7 ring closure involves the Mcm5 C-terminus and triggers Mcm4 ATP hydrolysis
Source: Nat Commun. 2025 Jan 2;16:14. doi: 10.1038/s41467-024-55479-1 (PMC11695723; doi:10.1038/s41467-024-55479-1)
Supplement: Supplementary file 1 — Supplementary Information [file 41467_2024_55479_MOESM1_ESM.pdf]

SUPPLEMENTARY INFORMATION

MCM2-7 ring closure involves the Mcm5 C-terminus and triggers Mcm4 ATP hydrolysis

Sarah V. Faull[1*], Marta Barbon[1,2*], Audrey Mossler[1], Zuanning Yuan[3], Lin Bai[3,a], L. Maximilian Reuter[1,b], Alberto Riera[1], Christian Winkler[1], Indiana Magdalou[1], Matthew Peach[1], Huilin Li[3#] and Christian Speck[1,2#]

[1] DNA Replication Group, Institute of Clinical Science, Imperial College London, London, W12 0NN, UK.

[2] MRC London Institute of Medical Sciences, Du Cane Road, London, W12 0NN, UK.

[3] Structural Biology Program, Van Andel Research Institute, Grand Rapids, MI 49503.

[a]Current address: Department of Biophysics, School of Basic Medical Sciences, Peking University, Beijing 100083, China

[b] Current address: Institute of Molecular Biology (IMB) gGmbH, Ackermannweg 4, 55128 Mainz, Germany

[*] Shared first authors

[#] Correspondence: Huilin.Li@vai.org; chris.speck@imperial.ac.uk

This files contains Supplementary Figures 1 to 15 and Supplementary Table 1.

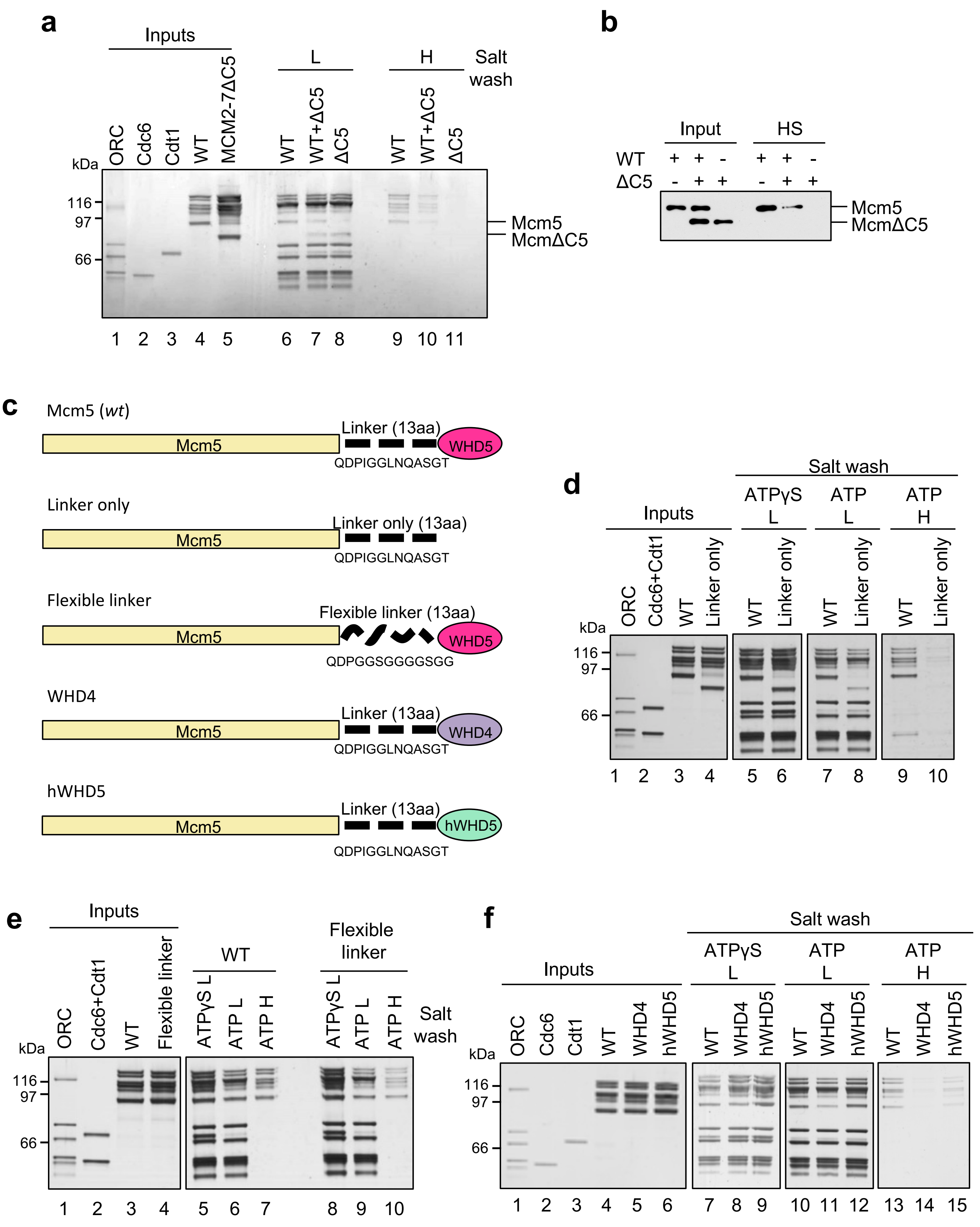
a
Inputs
L
H
Salt wash
ORC
Cdc6
Cdt1
WT
MCM2-7ΔC5
WT
WT+ΔC5
ΔC5
WT
WT+ΔC5
ΔC5
kDa
116
97
66
Mcm5
McmΔC5
1 2 3 4 5 6 7 8 9 10 11
b
Input
HS
WT + + - + + -
ΔC5 - + + - + +
Mcm5
McmΔC5
c
Mcm5 (wt)
Mcm5
Linker (13aa)
WHD5
QDPIGGLNQASGT
Linker only
Mcm5
Linker only (13aa)
QDPIGGLNQASGT
Flexible linker
Mcm5
Flexible linker (13aa)
WHD5
QDPGGSGGGGSGG
WHD4
Mcm5
Linker (13aa)
WHD4
QDPIGGLNQASGT
hWHD5
Mcm5
Linker (13aa)
hWHD5
QDPIGGLNQASGT
d
Salt wash
Inputs
ATPγS L
ATP L
ATP H
ORC
Cdc6+Cdt1
WT
Linker only
WT
Linker only
WT
Linker only
WT
Linker only
kDa
116
97
66
1 2 3 4 5 6 7 8 9 10
e
Inputs
WT
Flexible linker
ORC
Cdc6+Cdt1
WT
Flexible linker
ATPγS L
ATP L
ATP H
ATPγS L
ATP L
ATP H
Salt wash
kDa
116
97
66
1 2 3 4 5 6 7 8 9 10
f
Salt wash
Inputs
ATPγS L
ATP L
ATP H
ORC
Cdc6
Cdt1
WT
WHD4
hWHD5
WT
WHD4
hWHD5
WT
WHD4
hWHD5
WT
WHD4
hWHD5
kDa
116
97
66
1 2 3 4 5 6 7 8 9 10 11 12 13 14 15

Supplementary Figure 1. The Mcm5 winged helix domain is essential for pre-RC formation

(**a**) Pre-RC assembly using WT MCM2-7, MCM2-7-ΔC5 or a mixed population of WT MCM2-7 and MCM2-7-ΔC5 (1:1 ratio), followed by low (L) or high salt (H) washes. WT MCM2-7 is unable to promote the loading of the mutant MCM2-7-ΔC5 complex into salt-stable MCM2-7 double hexamers. (**b**) Same as (**a**), but only the high salt washes were analysed by western blot using an anti-Mcm5 antibody[48]. (**c**) Schematic representation of the mutants generated in the Mcm5 C-terminus (C5) in the context of the full-length MCM2-7. C5 consists of a winged-helix domain (WHD, residues 708-775) that is connected to the main body via a flexible linker (residues 693-707). Mutant constructs lacking the WHD domain, "linker only", and one where the linker was replaced with a random "flexible linker" sequence were generated. Additionally, the linker region was maintained and the WHD5 replaced with either the WHD of Mcm4 (WHD4) or a humainsed version of WHD5 (hWHD5). (**d**) Pre-RC assemblies of the linker only construct. The high salt wash shows that the "linker only" construct fails to form an MCM2-7 double hexamer. (**e**) Pre-RC assay of the flexible linker construct, showing that the protein can load double hexamers. (**f**) Pre-RC assay of constructs where WHD5 has been substituted with either WHD4 or hWHD5. Only the hWHD5 construct shows some helicase loading activity.

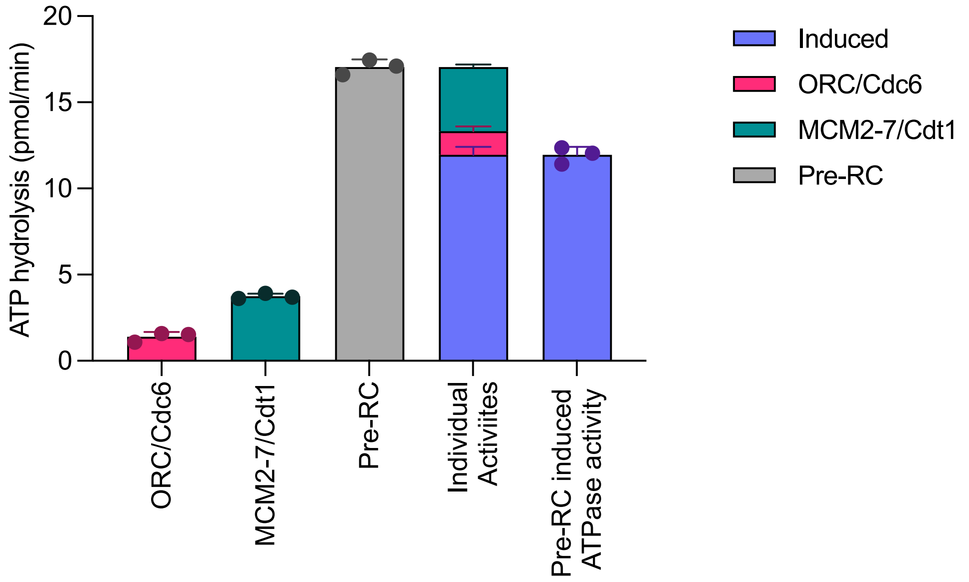

Supplementary Figure 2. Defining induced pre-RC activity

ATP hydrolysis rates were determined for the indicated proteins in the presence of ARS1 origin DNA using a radioactive ATPase assay. In the presence of all pre-RC proteins (ORC, Cdc6, Cdt1 and MCM2-7) a strong ATPase activity is induced (grey bar). The pre-RC induced ATPase activity (purple) is calculated by subtracting the values for the ORC/Cdc6 (pink bar) and MCM2-7-Cdt1 (green bar) components from the pre-RC value. n=3 biological replicates, represented as the mean ±SD. Source data are provided as a Source Data file.

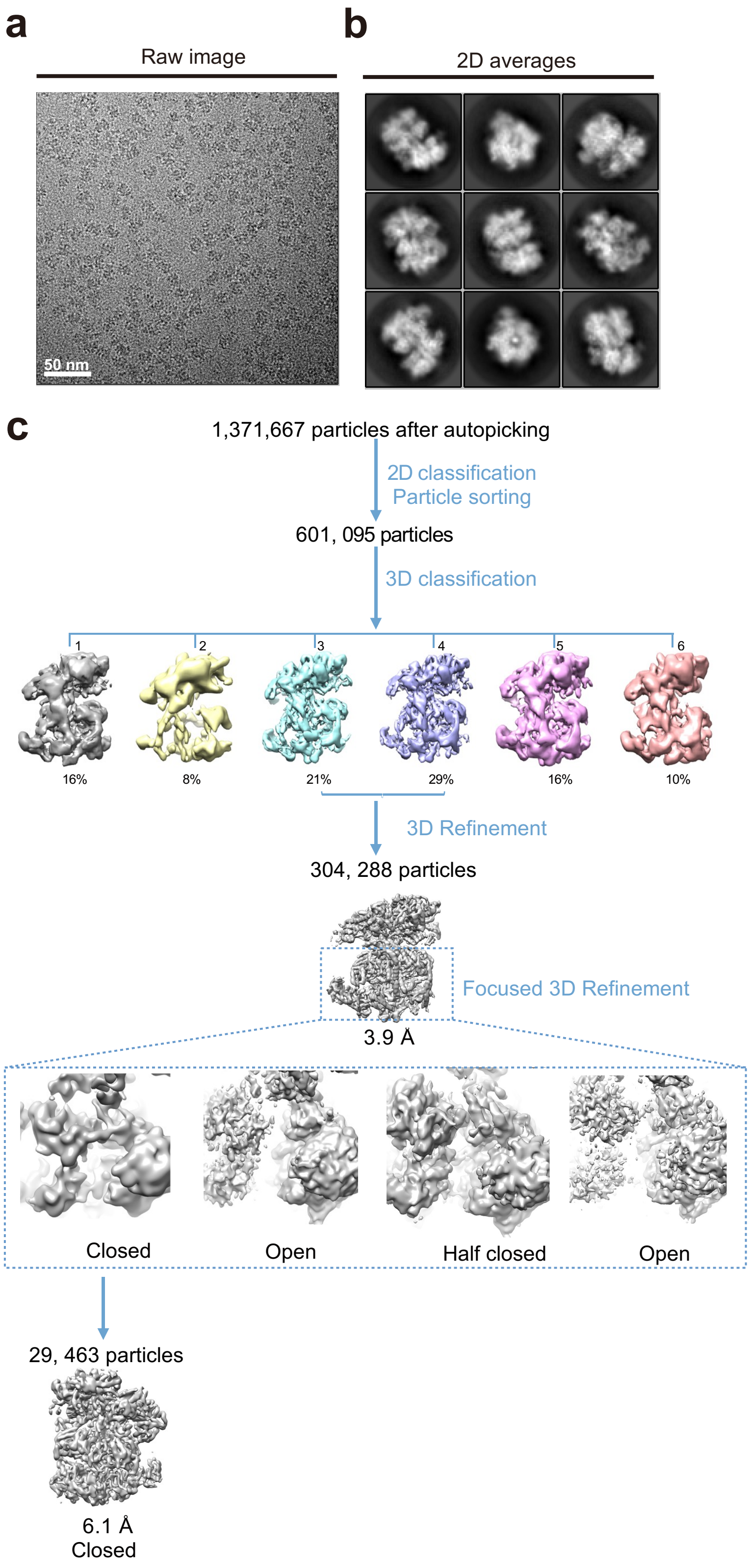
a
Raw image
50 nm
b
2D averages
c
1,371,667 particles after autopicking
2D classification
Particle sorting
601, 095 particles
3D classification
1
2
3
4
5
6
16%
8%
21%
29%
16%
10%
3D Refinement
304, 288 particles
Focused 3D Refinement
3.9 Å
Closed
Open
Half closed
Open
29, 463 particles
6.1 Å
Closed

Supplementary Figure 3. Cryo-EM image processing and 3D-reconstruction procedure

(a) A representative motion-corrected raw image of the OCCM particles. (**b**) Selected 2D averages representing different views of the complex. (**c**) Autopicking was used to select raw particle images. These particles were first sorted by 2D classification (~600,000) particles. These particles were further sorted into six 3D classes, representing ~300,000 particles for final 3D refinement. From the refined 3.9 Å structure (open-gate OCCM, EMD-8540), focused classification was performed on the MCM2-7 ring to obtain the closed-gate OCCM. The final 3D map of the closed-gate had an estimated resolution of 6.1 Å. This process also yielded two open-gate structures and a half-closed conformation.

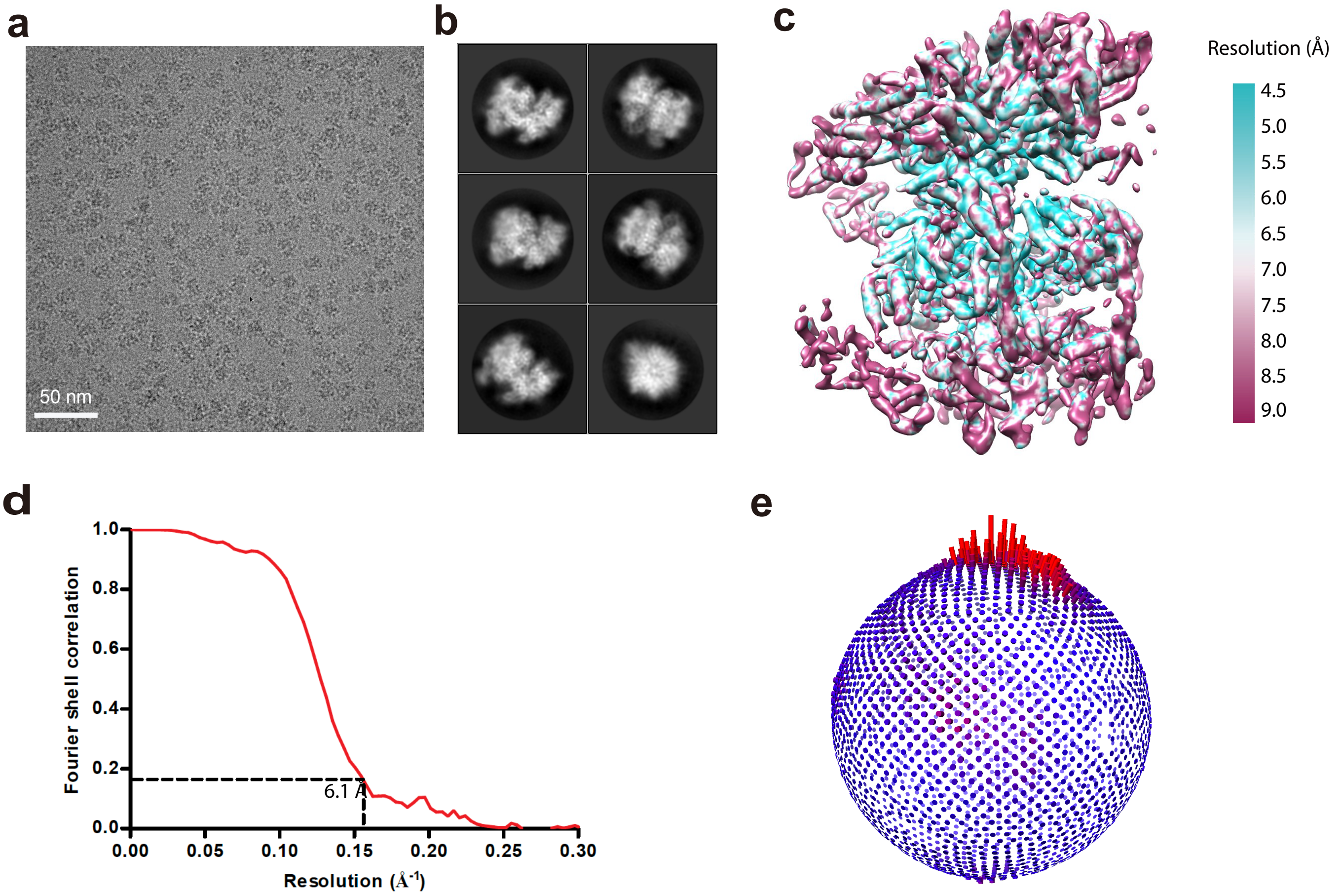

Supplementary Figure 4. Cryo-EM image processing and 3D-reconstruction procedure

(**a**) A representative motion-corrected micrograph of the OCCM particles. (**b**) Selected 2D class averages representing different views of the complex. (**c**) EM density map of the closed-gate OCCM coloured according to local resolution. (**d**) Fourier shell correlation (FSC) curve for the closed-gate OCCM. (**e**) Angular distribution of particle orientations.

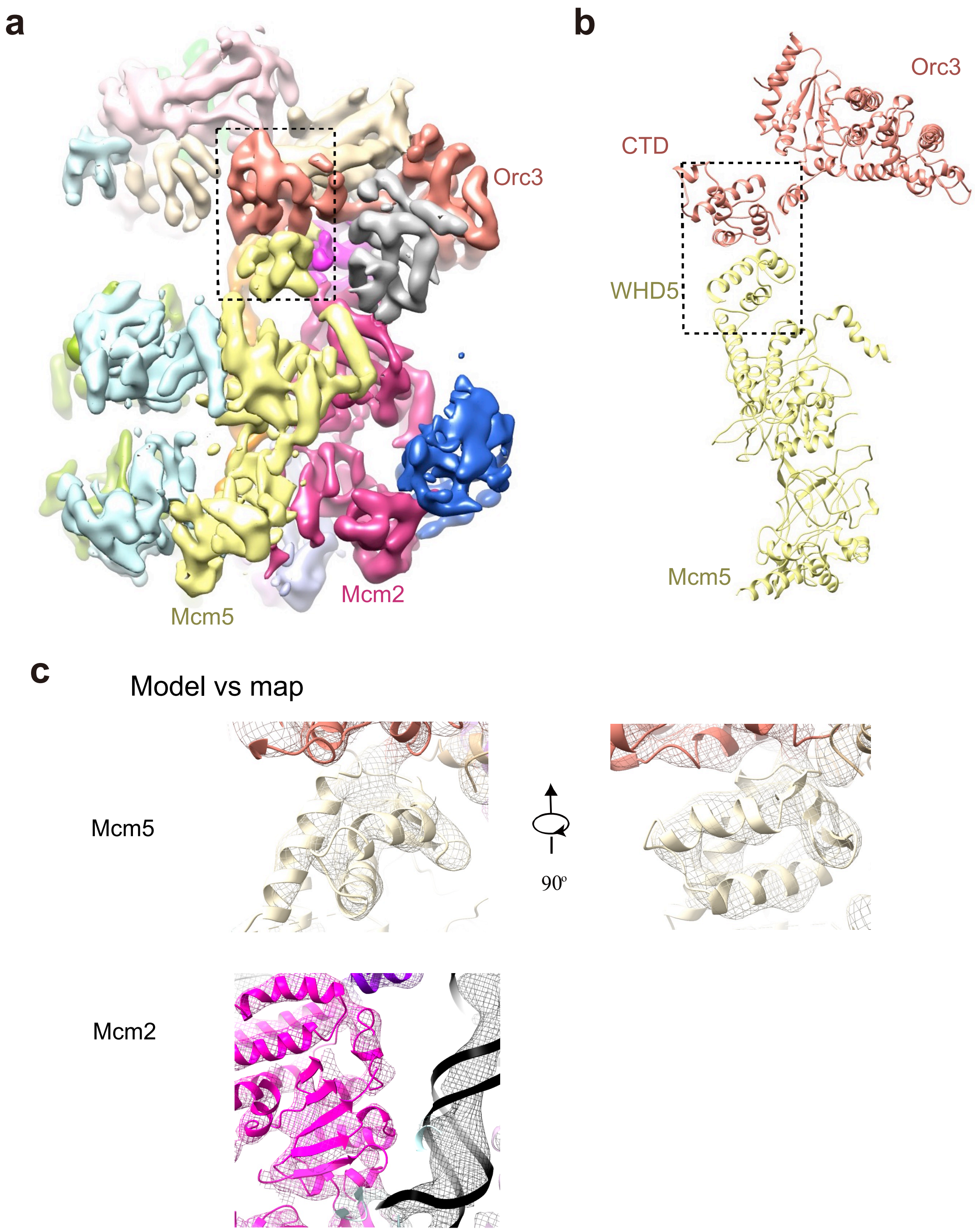

Supplementary Figure 5. Highlighting structural details of the closed-gate OCCM map

(a) The closed-gate OCCM stabilises the interaction between Mcm5 (yellow) and Orc3 (pink, dashed box), allowing density for the C-terminus of Mcm5 to be obtained. **(b)** The winged-helix domain of Mcm5 (WHD5) can be modelled for the first time in the context of the OCCM due to its interaction with the C-terminal domain (CTD) of Orc3. **(c)** Model-versus-map fits of the WHD5 and Mcm2 showing fitting of secondary structure elements into the density. The fitting of Mcm2 also shows its interaction with DNA.

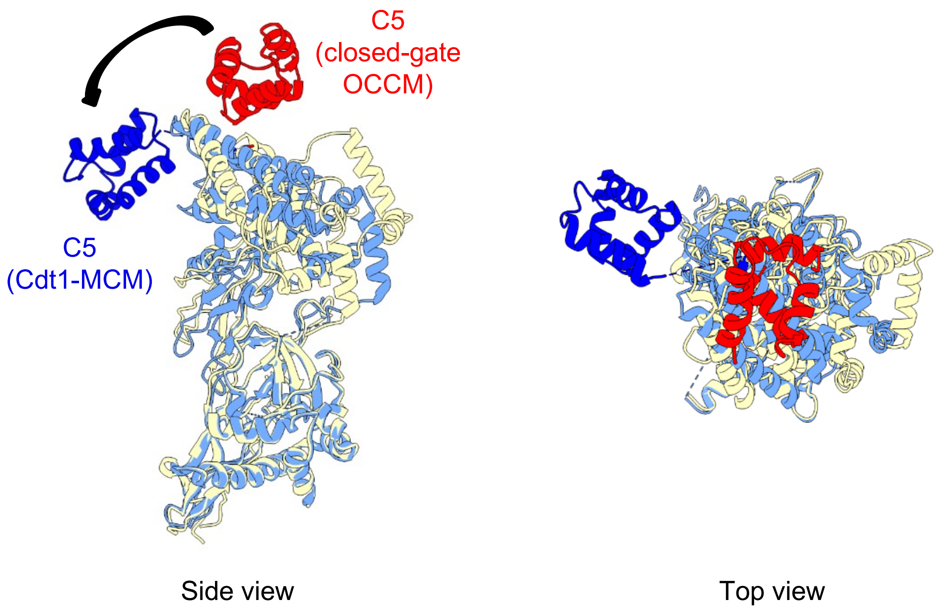

Supplementary Figure 6. The position of the Mcm5 C-terminus is altered in DNA-containing structures

The Mcm5 C-terminus (C5, royal blue) adopts a central position in the structure of MCM2-7 in complex with Cdt1 (5XF8, Mcm5 shown in pale blue). When DNA is present in the closed-gate OCCM (Mcm5 shown in pale yellow), C5 (red) is stacked on top of the AAA+ domain, away from the central DNA channel.

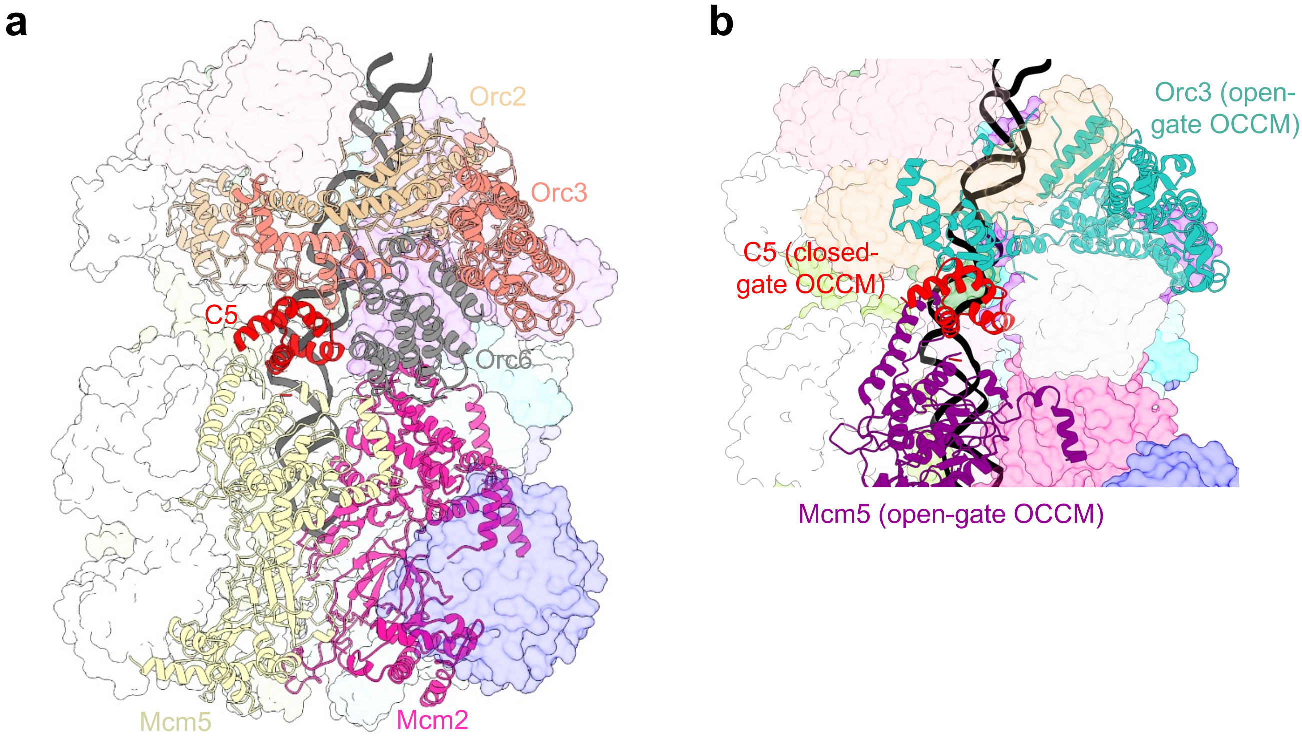

Supplementary Figure 7. Comparing the open- and closed- gate OCCM structures

(**a**) The new interactions between MCM2-7 and ORC that have been revealed by the closed-gate OCCM. The Mcm5 C-terminus (C5) is highlighted in red. In the closed-gate OCCM, C5 was observed next to Orc3 and the AAA+ domain of Mcm5 and in close proximity to the Orc2 N-terminal section. The subunits involved in interactions are displayed in cartoon form, with the other subunits displayed in transparent surface view. (**b**) The Orc3-C5 interaction observed in the closed-gate OCCM is sterically hindered in the context of the open-gate OCCM complex. C5 from the closed structure is shown in red in a position that clashes with the Mcm5 AAA+ domain (purple) and Orc3 (green) from the open-ringed structure (PDB 5V8F)[14]. This provides a structural explanation as to why C5 can only interact when the Mcm2/Mcm5 gate is closed.

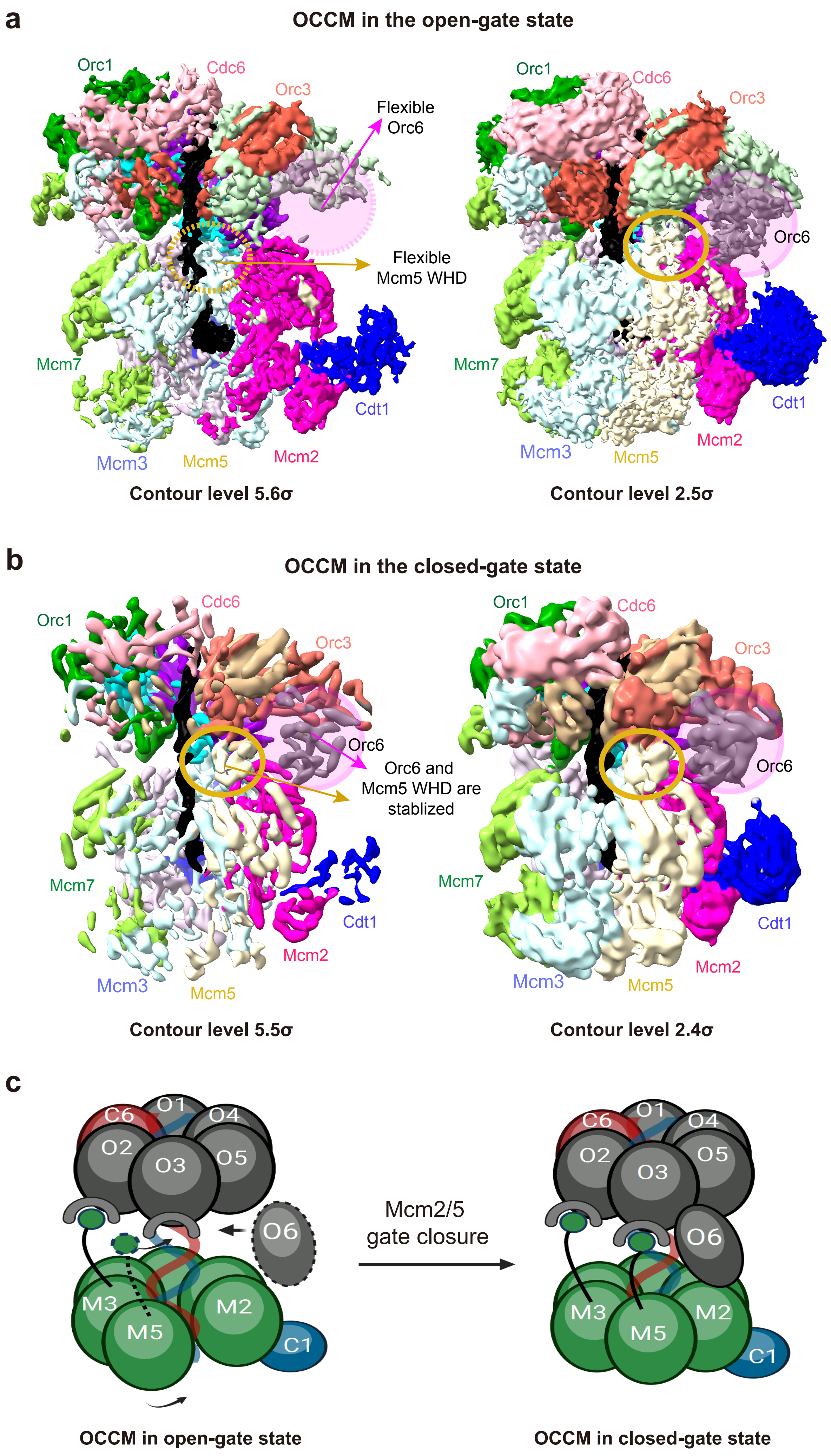
a
OCCM in the open-gate state
Orc1
Cdc6
Orc3
Flexible
Orc6
Flexible
Mcm5 WHD
Mcm7
Cdt1
Mcm3
Mcm5
Mcm2
Contour level 5.6σ
Orc1
Cdc6
Orc3
Orc6
Mcm7
Cdt1
Mcm3
Mcm5
Mcm2
Contour level 2.5σ
b
OCCM in the closed-gate state
Orc1
Cdc6
Orc3
Orc6
Orc6 and
Mcm5 WHD are
stablized
Mcm7
Cdt1
Mcm3
Mcm5
Mcm2
Contour level 5.5σ
Orc1
Cdc6
Orc3
Orc6
Mcm7
Cdt1
Mcm3
Mcm5
Mcm2
Contour level 2.4σ
c
C6
O1
O4
O2
O3
O5
O6
M3
M5
M2
C1
Mcm2/5
gate closure
OCCM in open-gate state
OCCM in closed-gate state

Supplementary Figure 8. Comparison of the OCCM cryo-EM maps in the previously published open-gate state and the current closed-gate state

(**a**) The EM map of open-gate OCCM (PDB ID 5V8F) is shown in a high threshold (5.6σ, left) and a low threshold (2.5σ, right). Mcm5 and Orc6 are visible only at the low threshold due to their flexibility. (**b**) The EM map of the closed-gate OCCM (this study) is displayed at a high threshold (5.5σ, left) and a low threshold (2.4σ, right) threshold. Both Mcm5 and Orc6 are visible at the high threshold, indicating their stabilisation upon gate closure. (**c**) A cartoon representation illustrating transition from the open-gate model (PDB ID 5V8F), and the closed-gate OCCM models (this study). M2, M3, and M5 represent Mcm2, Mcm3, and Mcm5, respectively. O1-6 stands for Orc1 through Orc6. C6 is abbreviation for Cdc6, and C1 is abbreviation for Cdt1.

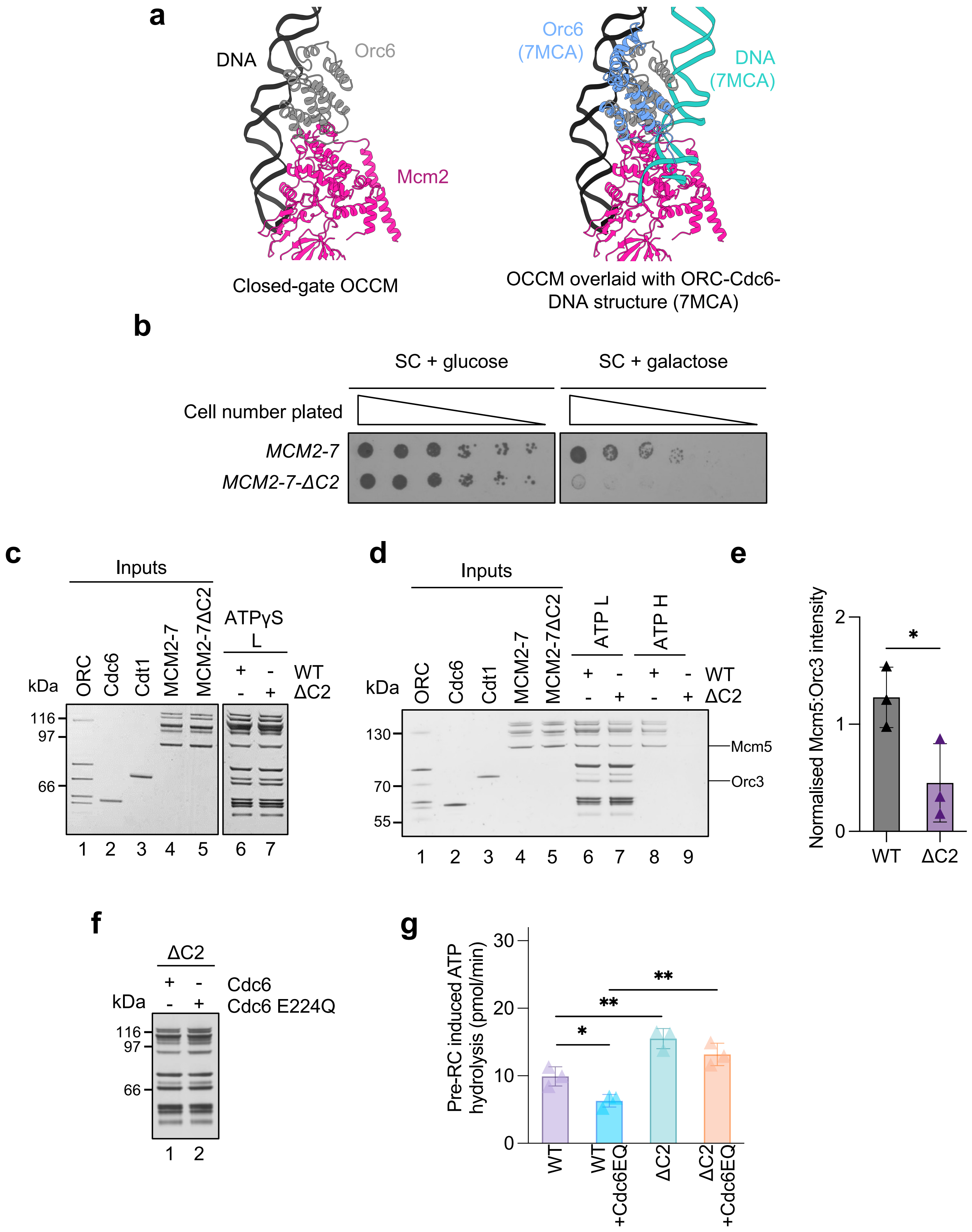
a
DNA
Orc6
Mcm2
Closed-gate OCCM
Orc6 (7MCA)
DNA (7MCA)
OCCM overlaid with ORC-Cdc6-DNA structure (7MCA)
b
SC + glucose
SC + galactose
Cell number plated
MCM2-7
MCM2-7-ΔC2
c
Inputs
ORC
Cdc6
Cdt1
MCM2-7
MCM2-7ΔC2
ATPγS L
WT
ΔC2
kDa
116
97
66
1 2 3 4 5 6 7
d
Inputs
ATP L
ATP H
Mcm5
Orc3
130
70
55
1 2 3 4 5 6 7 8 9
e
Normalised Mcm5:Orc3 intensity
WT
ΔC2
f
ΔC2
Cdc6
Cdc6 E224Q
g
Pre-RC induced ATP hydrolysis (pmol/min)
WT
WT +Cdc6EQ
ΔC2
ΔC2 +Cdc6EQ

Supplementary Figure 9. Exploring the role of the Mcm2 C-terminus in pre-RC formation

(**a**) Cartoon from the closed-gate OCCM showing the interaction between the N-terminus of Orc6 and the Mcm2 AAA+ domain. (**b**) Dominant lethality of WT MCM2-7 and MCM2-7-ΔC2 was tested by plating 5-fold dilutions onto selective dropout plates containing either 2% glucose or 2% galactose. (**c** and **d**) Silver-stained gels of pre-RC assay with WT MCM2-7 and MCM2-7-ΔC2 in the presence of ATPγS or ATP followed by low salt (L) or high salt (H) washes. **(e)** Normalised Mcm5:Orc3 band intensity for WT MCM2-7 and MCM2-7-ΔC2 for low salt washed reactions from part (d). Data is represented as the mean of three biological, error bars represent standard deviation. Analysis was performed using a two-tailed t-test, * P=0.0401 (**f**) Low salt wash pre-RC assay with WT MCM2-7 and MCM2-7-ΔC2 in the presence of either Cdc6 or Cdc6 E224Q. (**g**) Pre-RC induced ATPase activities of WT MCM2-7 and MCM2-7-ΔC2 in the presence of either WT Cdc6 or Cdc6 E224Q. Means were calculated from three biological repeats and error bars represent the standard deviation. Analysis was performed using a two-tailed t-test, * P=0.0215, ** P=0.0093 and P=0.0033.

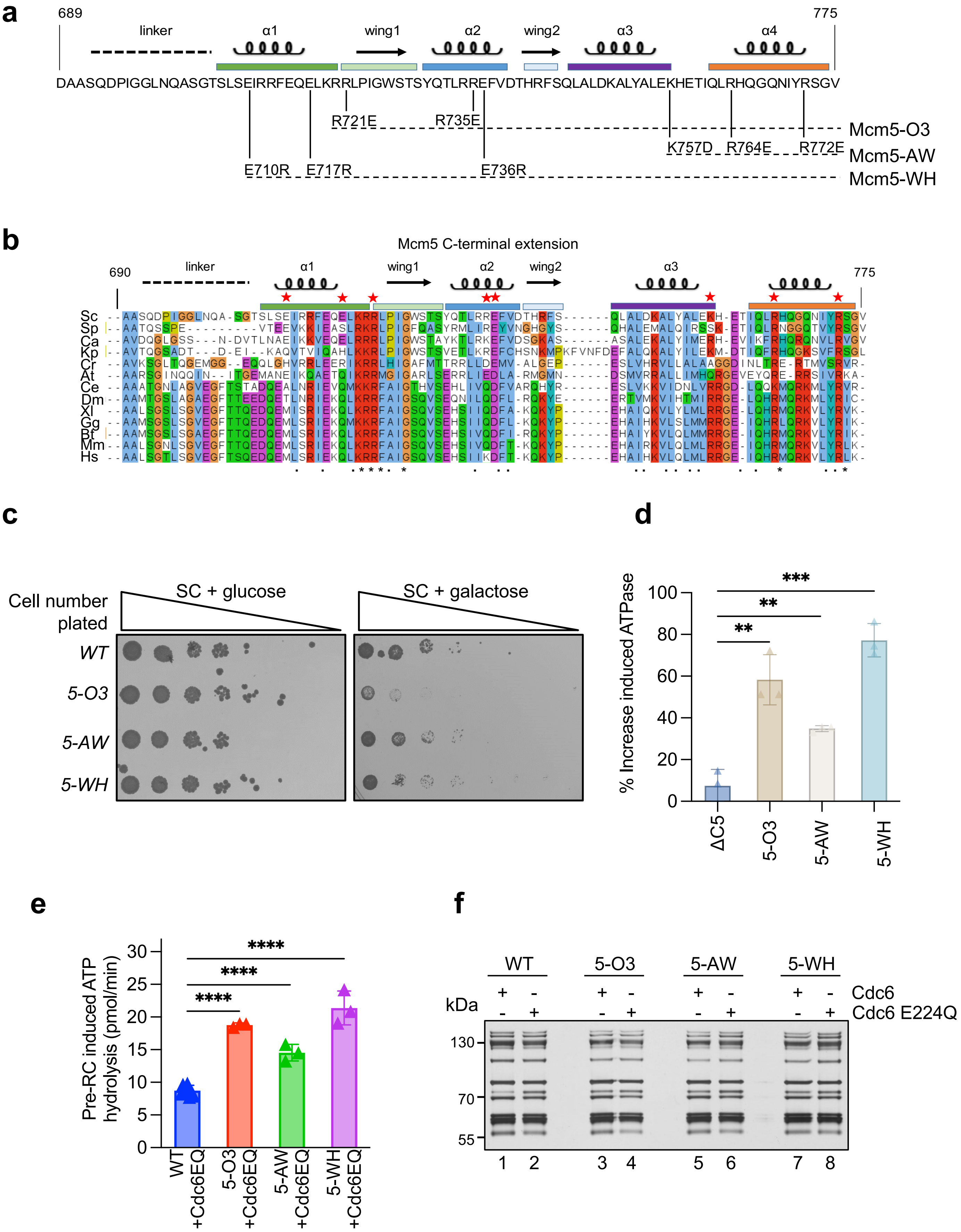

a
689
775
linker
α1
wing1
α2
wing2
α3
α4
DAASQDPIGGLNQASGTSLSEIRRFEQELKRRLPIGWSTSYQTLRREFVDTHRFSQLALDKALYALEKHETIQLRHQGQNIYRSGV
R721E
R735E
Mcm5-O3
K757D
R764E
R772E
Mcm5-AW
E710R
E717R
E736R
Mcm5-WH
b
Mcm5 C-terminal extension
690
775
linker
α1
wing1
α2
wing2
α3
Sc
Sp
Ca
Kp
Cr
At
Ce
Dm
Xl
Gg
Bt
Mm
Hs
c
Cell number plated
SC + glucose
SC + galactose
WT
5-O3
5-AW
5-WH
d
% Increase induced ATPase
100
80
60
40
20
0
***
**
**
ΔC5
5-O3
5-AW
5-WH
e
Pre-RC induced ATP hydrolysis (pmol/min)
30
25
20
15
10
5
0
****
****
****
WT +Cdc6EQ
5-O3 +Cdc6EQ
5-AW +Cdc6EQ
5-WH +Cdc6EQ
f
WT
5-O3
5-AW
5-WH
kDa
Cdc6
Cdc6 E224Q
130
70
55
1
2
3
4
5
6
7
8

Supplementary Figure 10. The location, conservation and effects of mutations in the Mcm5 C-terminus

(**a**) The domain organisation of the Mcm5 C-terminus (amino acids 689-775). The four alpha helices (α1 in green, α2 in blue, α3 in purple, α4 in orange) and two wings (pale green and pale blue) are indicated above the sequence. The residues that have been mutated in the constructs Mcm5-AW, Mcm5-WH and Mcm5-O3 are shown by vertical lines. (**b**) Alignment of Mcm5 C-terminal extensions along with the secondary structure of *S. cerevisiae* Mcm5. Red stars indicate the mutation sites shown in part a. (**c**) Dominant lethality assay of WT *Mcm5* and mutants were tested by plating 5-fold dilutions onto selective dropout plates containing either 2% glucose or 2% galactose. (**d**) Percentage change of MCM2-7-ΔC5 and Mcm5 mutants compared to *wt* MCM2-7 control in their respective experiments. Means are calculated from three biological repeats, error bars represent standard deviation. Mutants were compared to MCM2-7-ΔC5 using a two-tailed t-test. ** P=0.0036 and P=0.040 and *** P=0.0004. (**e**) Induced ATP hydrolysis of Mcm5 mutants when assayed with Cdc6 E224Q. Values are compared to the hydrolysis rates observed with the induced pre-RC rates of the Mcm5 mutants with WT Cdc6. Means were calculated from three biological repeats and error bars represent the standard deviation. Analysis was performed using a two-tailed t-test ****P=<0.0001. (**f)** Pre-RC assay of WT MCM2-7 or Mcm5 mutants under low salt conditions in the presence or absence of Cdc6 or the Cdc6 E224Q ATPase mutant. Source data are provided as a Source Data file.

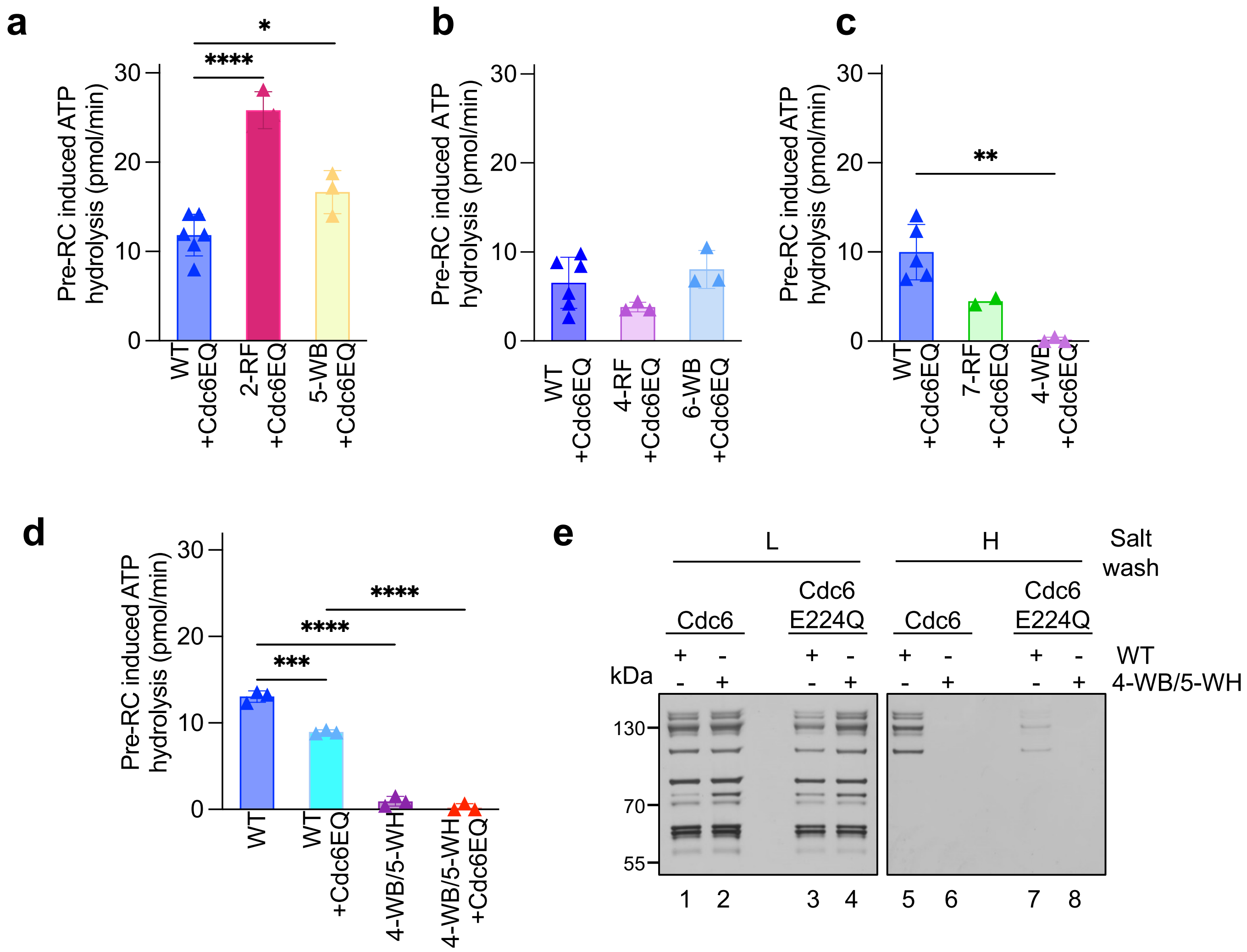
a
b
c
d
e
Pre-RC induced ATP hydrolysis (pmol/min)
WT +Cdc6EQ
2-RF +Cdc6EQ
5-WB +Cdc6EQ
4-RF +Cdc6EQ
6-WB +Cdc6EQ
7-RF +Cdc6EQ
4-WB +Cdc6EQ
WT
4-WB/5-WH
4-WB/5-WH +Cdc6EQ
L
H
Salt wash
Cdc6
Cdc6 E224Q
WT
4-WB/5-WH
kDa
130
70
55
1
2
3
4
5
6
7
8

Supplementary Figure 11. Characterisation of MCM2-7 ATPase mutants in complex with the Cdc6 E224Q ATPase mutant

(**a**) Pre-RC induced ATPase activities of WT MCM2-7, Mcm2 arginine finger mutant (Mcm2-RF) and Mcm5 Walker B mutant (Mcm5-WB) in the presence of Cdc6 E224Q. Means were calculated from three biological repeats for mutants and n=6 for WT. Error bars represent standard deviation. Analysis was performed using a two-tailed t-test, **** P=<0.0001, *P=0.0235. (**b**) Pre-RC induced ATPase activities of WT MCM2-7, Mcm4 arginine finger mutant (Mcm4-RF) and Mcm6 Walker B mutant (Mcm6-WB) in the presence of Cdc6 E224Q. Means were calculated from three biological repeats for mutants and n=6 for WT and error bars represent standard deviation. (**c**) Pre-RC induced ATPase activities of WT MCM2-7, Mcm7 arginine finger mutant (Mcm7-RF) and Mcm4 Walker B mutant (Mcm4-WB) in the presence of Cdc6 E224Q. Means were calculated from three biological repeats for Mcm4-WB, n=2 for 7-RF and n=5 for WT. Error bars represent standard deviation. Analysis was performed using a two-tailed t-test, **P=0.0019. (**d**) Pre-RC induced ATPase activities of WT MCM2-7 and a double mutant of Mcm4 Walker B and Mcm5-WH (Mcm4-WB/5-WH) in the presence of either Cdc6 or Cdc6 E224Q. Values for Mcm4-WB/5-WH in complex with Cdc6 E224Q were too low to distinguish from background (DNA only) controls. Means were calculated from three biological repeats and error bars represent the standard deviation. Analysis was performed using a two-tailed t-test,*** P=0.0005, **** P=<0.0001. (**e**) Pre-RC assembly of WT MCM2-7 and Mcm4-WB/5-WH. The reactions were assembled in ATP or ATPγS and washed with low salt buffer. Source data are provided as a Source Data file.

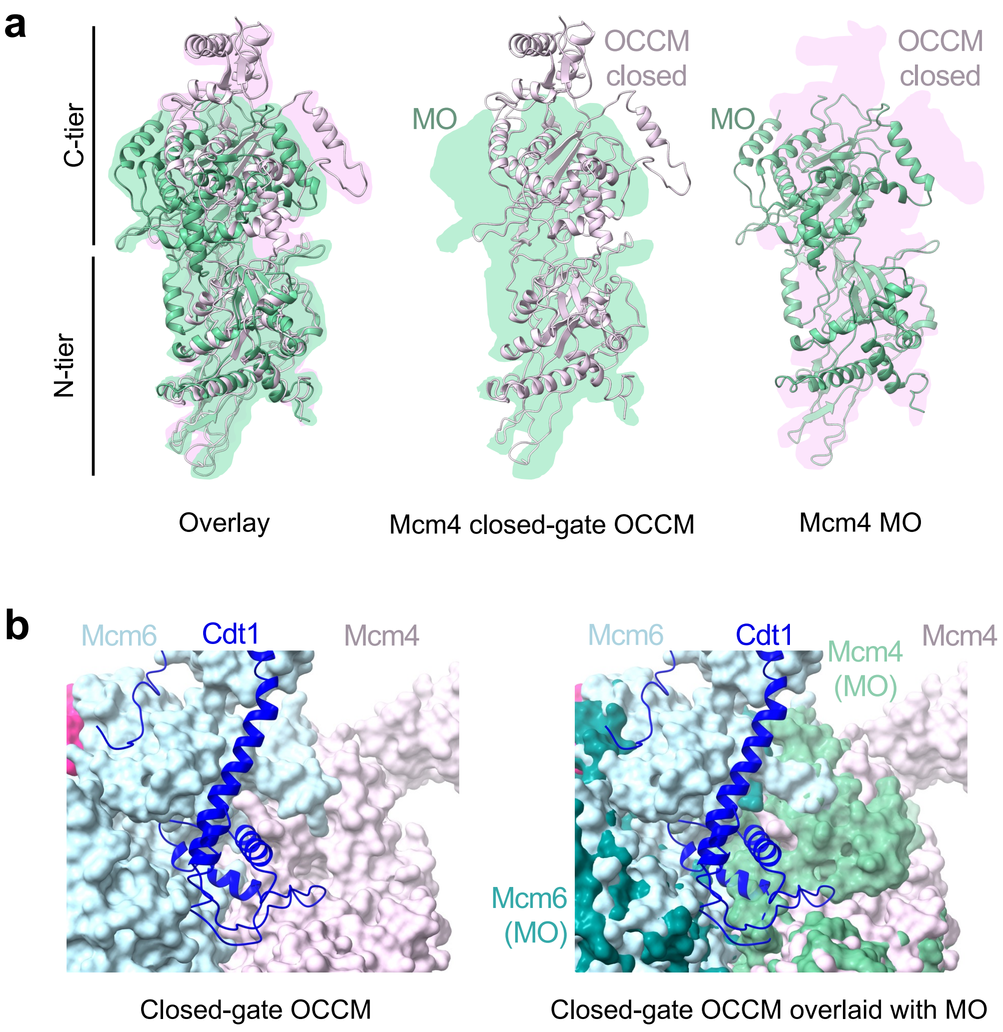

Supplementary Figure 12. Overlaying the structures of the MO and the closed-gate OCCM provides a structural explanation for Cdt1 release

(**a**) Comparison of Mcm4 in the pre- (lilac, closed-gate OCCM) and post- (green, MO (PDB ID 6RQC)) ATP hydrolysis states. The N-tier of the Mcm ring remains fixed, but major topological changes occur in the C-tier. (**b**) In the closed-gate OCCM , the C-terminus of Cdt1 (royal blue) interacts with Mcm4 (lilac) at its interface with Mcm6 (light blue). In the next loading intermediate in the pre-RC pathway, MO (PDB ID 6RQC, green), this pocket is disrupted, leading to a spatial clash between the two structures (right). The rearrangement of this interface will support the release of Cdt1 from the OCCM complex.

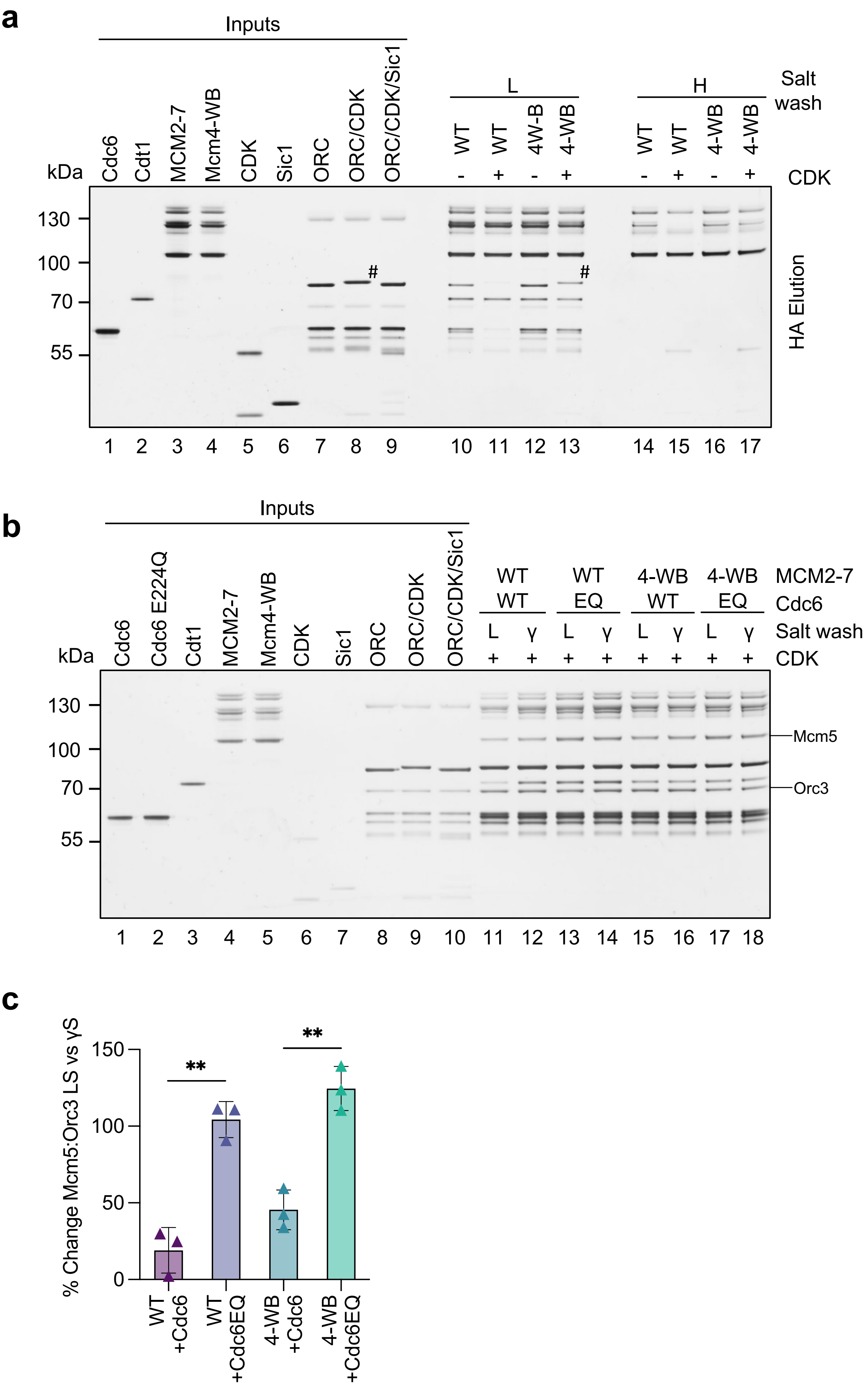

a
Inputs
kDa
Cdc6
Cdt1
MCM2-7
Mcm4-WB
CDK
Sic1
ORC
ORC/CDK
ORC/CDK/Sic1
L
H
Salt wash
WT
WT
4W-B
4-WB
WT
WT
4-WB
4-WB
CDK
- + - + - + - +
130
100
70
55
#
#
HA Elution
1 2 3 4 5 6 7 8 9 10 11 12 13 14 15 16 17
b
Inputs
Cdc6
Cdc6 E224Q
Cdt1
MCM2-7
Mcm4-WB
CDK
Sic1
ORC
ORC/CDK
ORC/CDK/Sic1
WT WT 4-WB 4-WB MCM2-7
WT EQ WT EQ Cdc6
L γ L γ L γ L γ Salt wash
+ + + + + + + + CDK
kDa
130
100
70
55
Mcm5
Orc3
1 2 3 4 5 6 7 8 9 10 11 12 13 14 15 16 17 18
c
% Change Mcm5:Orc3 LS vs γS
150
100
50
0
**
**
WT +Cdc6
WT +Cdc6EQ
4-WB +Cdc6
4-WB +Cdc6EQ

Supplementary Figure 13. ORC phosphorylation induces MCM2-7 ring splitting in unproductive pre-RC complexes

(a) Lanes 1-4, 7: 15% inputs of proteins, lanes 5-6 100% protein inputs. Lane 8: ORC phosphorylated by CDK. Lane 9: pre-incubation of Sic1 inhibits ORC phosphorylation by CDK. Lanes 10-17: Low or high salt washes of pre-RC reactions assembled in solution, pulleddown via the HA-tag on Mcm3, washed and then eluted using HA peptide. In the lanes +CDK, ORC was phosphorylated for 20 minutes, then Sic1 was added to inhibit CDK prior to the addition of Cdc6, Cdt1 and MCM2-7 or the Mcm4-WB mutant. # denotes the visible shift of the Orc2 band due to phosphorylation. **(b)** Pre-RC assay of CDK-phosphorylated ORC with the MCM2-7 4-WB mutant and or Cdc6 E224Q (EQ), showing that the addition of Cdc6 E224Q provides similar levels of stabilisation between MCM2-7 WT and Mcm4-WB. Lanes 1-5, 8: 20% inputs of proteins, lanes 6-7 40% protein inputs. Lane 9: ORC phosphorylated by CDK. Lane 10: preincubation of Sic1 inhibits ORC phosphorylation by CDK. **(c)** Band densitometry of the ratio of ratio of Mcm5:Orc3 bands with conditions expressed as a percentage change of low salt washed lanes (LS) compared to lanes washed with ATPγS. n=3 biological repeats, error bars represent standard error. Analysis was performed using a two-tailed t-test ** P=0.0014 and P=0.0021. Source data are provided as a Source Data file.

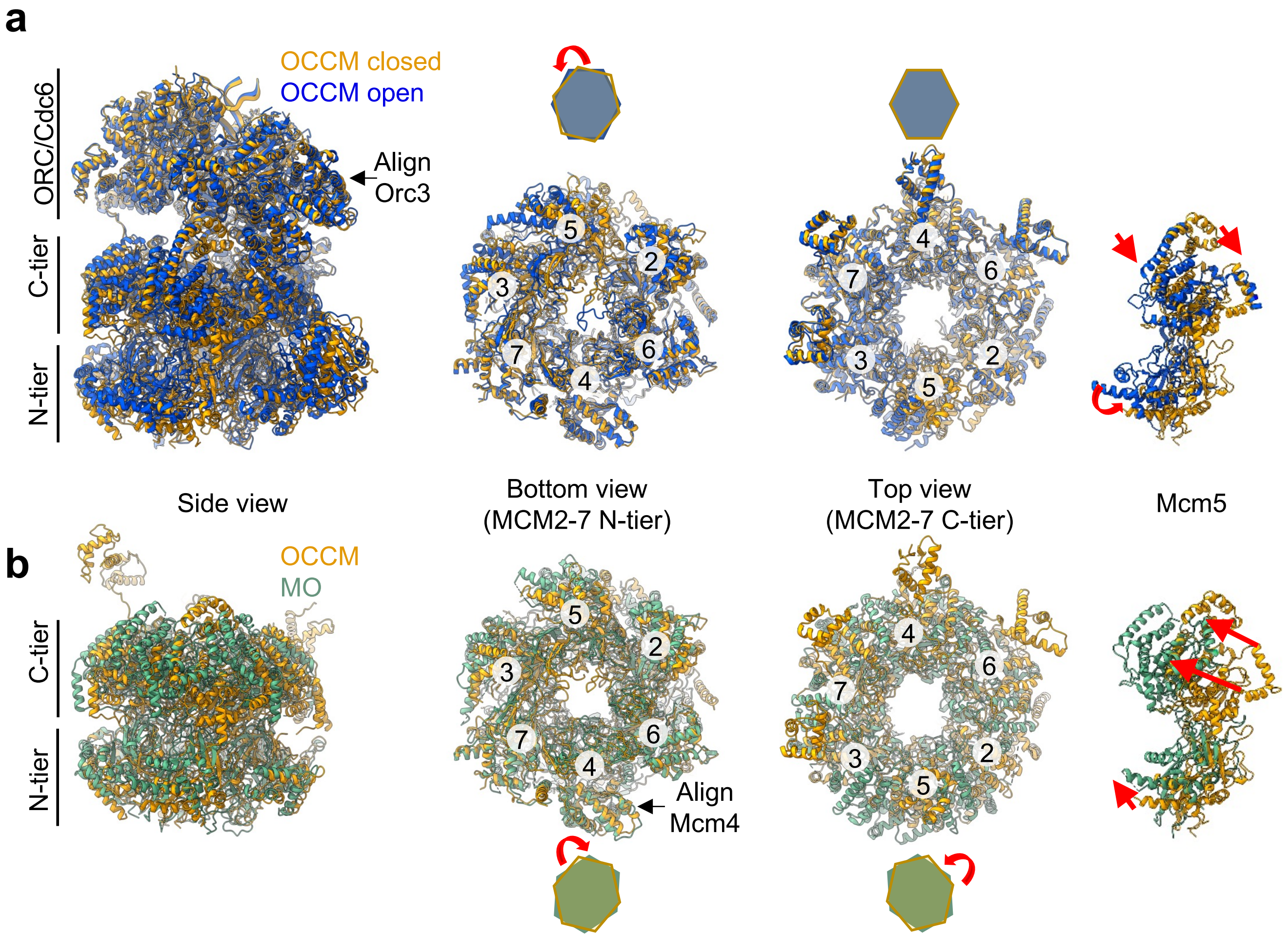

Supplementary Figure 14. MCM2-7 conformational changes

(a) Aligning the open- (PDB ID 5V8F, blue) and closed (yellow) structures by Orc3 reveals that the closure of the Mcm2/Mcm5 interface occurs through a rotation in the N-terminal domains of MCM2-7. This can be viewed via the Mcm5 subunit (far right), where the C-tier domain shows only a small compaction, but the lower (N-tier) part of the structure shows a large rotation. (**b**) Alignment of the Mcm subunits of the closed-gate OCCM (yellow) and MO (green, PDB ID 6RQC) via Mcm4 reveals significant torsion of the ring due to ATP hydrolysis and the release of loading factors Cdc6, Cdt1 and the “flipping” of ORC to the C-terminus of MCM2-7.

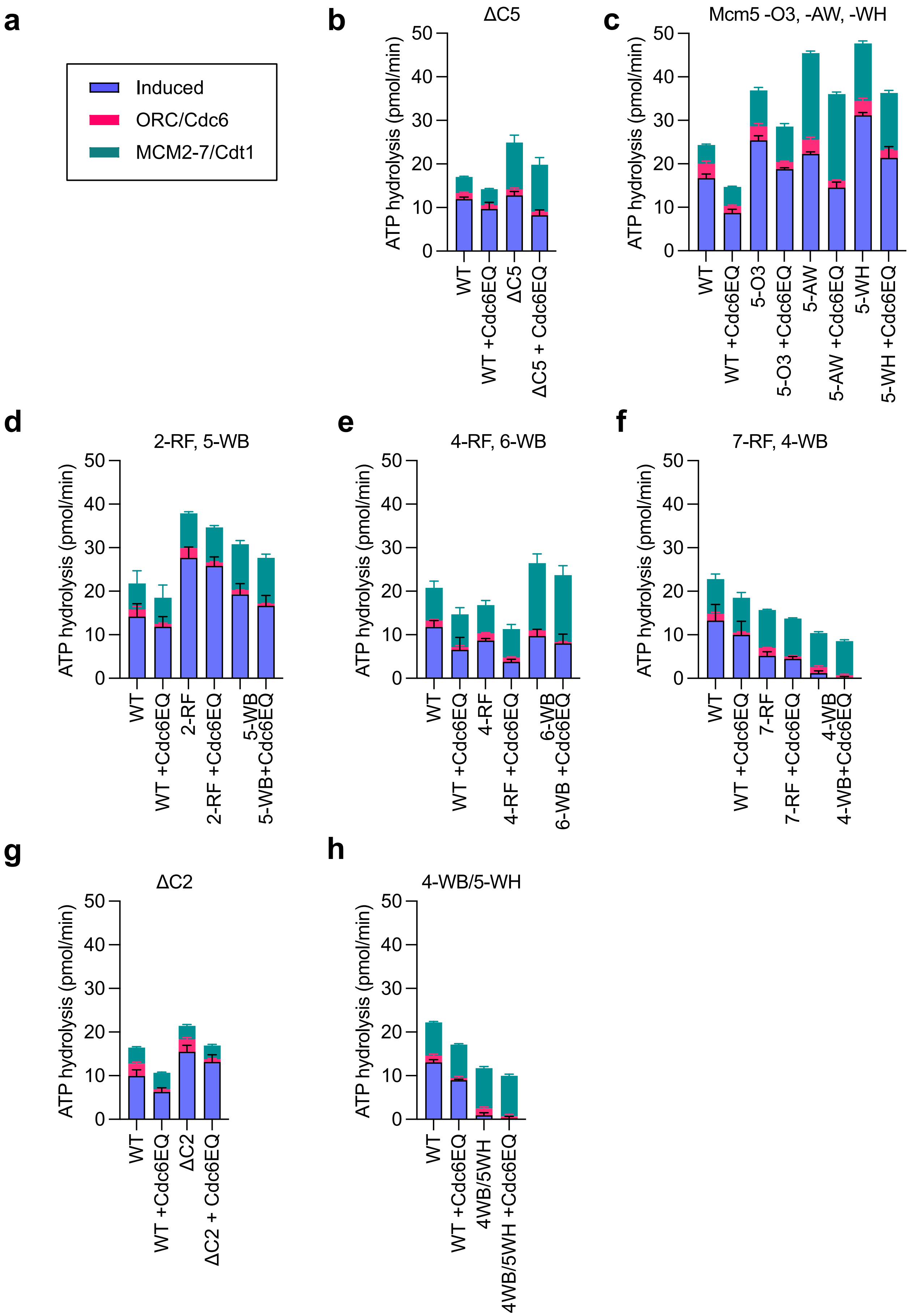
a
Induced
ORC/Cdc6
MCM2-7/Cdt1
b
ΔC5
ATP hydrolysis (pmol/min)
WT
WT +Cdc6EQ
ΔC5
ΔC5 + Cdc6EQ
c
Mcm5 -O3, -AW, -WH
WT
WT +Cdc6EQ
5-O3
5-O3 +Cdc6EQ
5-AW
5-AW +Cdc6EQ
5-WH
5-WH +Cdc6EQ
d
2-RF, 5-WB
WT
WT +Cdc6EQ
2-RF
2-RF +Cdc6EQ
5-WB
5-WB+Cdc6EQ
e
4-RF, 6-WB
4-RF
4-RF +Cdc6EQ
6-WB
6-WB +Cdc6EQ
f
7-RF, 4-WB
7-RF
7-RF +Cdc6EQ
4-WB
4-WB+Cdc6EQ
g
ΔC2
ΔC2
ΔC2 + Cdc6EQ
h
4-WB/5-WH
4WB/5WH
4WB/5WH +Cdc6EQ

Supplementary Figure 15. Individual ATPase activities of pre-RC proteins for all mutants

(a) Key defining the colour scheme used in parts b-h. Induced ATPase activity (blue) is the ATPase rate for the pre-RC complex minus the individual activities of Orc/Cdc6 (pink) and Mcm2-7/Cdt1 (green). The cumulative height of the bar represents the pre-RC activity. Data shown is representative of at least n=3, n=2 for 7-RF and corresponds to the induced activities represented in the other figures. (b) Mcm2-7-ΔC5. (c) Mcm5 mutants 5-O3, 5-AW and 5-WH. (d) Mcm2-RF and Mcm5-WB. (e) Mcm4-RF and Mcm6-WB (f) Mcm7-RF and Mcm4-WB. (g) MCM2-7-ΔC2. (h) Mcm4-WB/5-WH. Source data are provided as a Source Data file.

Supplementary Table 1. Cryo-EM data collection and refinement statistics.

**Data Collection**	**WT OCCM** (“closed-gate” conformer)
EM equipment	FEI Titan Krios
Voltage (kV)	300
Detector	Gatan K2
Pixel size (Å)	1.01
Electron dose ($e^-/Å^2$)	50
Under-focus range (μm)	1.5 – 2.5
**Reconstruction**	
Software	RELION 2.0
Number of used particles	29,463
Resolution (Å)	6.1
Map sharpening B-factor ($Å^2$)	-162
**Model composition**	
Peptide chains	14
Protein residues	7034
**R.m.s deviations**	
Bond length (Å)	0.007
Bond angle	1.35°
**Ramachandran plot**	
Preferred (%)	93.39
Allowed (%)	6.26
Outlier (%)	0.35
**Validation**	
Molprobity score	1.52 (95%)
Rotamer outliers (%)	0.75
