# Supplementary material for: MCM2-7 ring closure involves the Mcm5 C-terminus and triggers Mcm4 ATP hydrolysis
Source: Nat Commun. 2025 Jan 2;16:14. doi: 10.1038/s41467-024-55479-1 (PMC11695723; doi:10.1038/s41467-024-55479-1)
Supplement: Supplementary file 2 — Description of Additional Supplementary Files [file 41467_2024_55479_MOESM2_ESM.pdf]

Description of Additional Supplementary Files

File Name: Supplementary Data 1
Description: Reagents and resources
