# Supplementary material for: MCM2-7 ring closure involves the Mcm5 C-terminus and triggers Mcm4 ATP hydrolysis
Source: Nat Commun. 2025 Jan 2;16:14. doi: 10.1038/s41467-024-55479-1 (PMC11695723; doi:10.1038/s41467-024-55479-1)
Supplement: Supplementary file 4 — Reporting Summary [file 41467_2024_55479_MOESM4_ESM.pdf]

nature portfolio

Corresponding author(s): Christian Speck and Hulin Li

Last updated by author(s): Oct 28, 2024

Reporting Summary

Nature Portfolio wishes to improve the reproducibility of the work that we publish. This form provides structure for consistency and transparency in reporting. For further information on Nature Portfolio policies, see our Editorial Policies and the Editorial Policy Checklist.

Statistics

For all statistical analyses, confirm that the following items are present in the figure legend, table legend, main text, or Methods section.

n/a	Confirmed	
☐	☒	The exact sample size (*n*) for each experimental group/condition, given as a discrete number and unit of measurement
☐	☒	A statement on whether measurements were taken from distinct samples or whether the same sample was measured repeatedly
☐	☒	The statistical test(s) used AND whether they are one- or two-sided<br>*Only common tests should be described solely by name; describe more complex techniques in the Methods section.*
☐	☒	A description of all covariates tested
☐	☒	A description of any assumptions or corrections, such as tests of normality and adjustment for multiple comparisons
☐	☒	A full description of the statistical parameters including central tendency (e.g. means) or other basic estimates (e.g. regression coefficient) AND variation (e.g. standard deviation) or associated estimates of uncertainty (e.g. confidence intervals)
☐	☒	For null hypothesis testing, the test statistic (e.g. *F*, *t*, *r*) with confidence intervals, effect sizes, degrees of freedom and *P* value noted<br>*Give P values as exact values whenever suitable.*
☒	☐	For Bayesian analysis, information on the choice of priors and Markov chain Monte Carlo settings
☒	☐	For hierarchical and complex designs, identification of the appropriate level for tests and full reporting of outcomes
☒	☐	Estimates of effect sizes (e.g. Cohen's *d*, Pearson's *r*), indicating how they were calculated

Our web collection on statistics for biologists contains articles on many of the points above.

Software and code

Policy information about availability of computer code

Data collection	FEI Titan Krios G2 electron microscope, Gatan K2 direct electron detector
Data analysis	Relion 2.0, UCSF Chimera 1.17, UCSF ChimeraX v1.7, Coot 0.9.8, SWISS-MODEL (https://swissmodel.expasy.org/), Phenix 1.14, MolProbity 4.4, PyMOL Molecular Graphics System (Version 2.0 Schrödinger, LLC), DiscoverMP software (v. 2023R2, Refeyn Ltd), Multi Gauge V2.3, GraphPadPrism v9

For manuscripts utilizing custom algorithms or software that are central to the research but not yet described in published literature, software must be made available to editors and reviewers. We strongly encourage code deposition in a community repository (e.g. GitHub). See the Nature Portfolio guidelines for submitting code & software for further information.

Data

Policy information about availability of data

All manuscripts must include a data availability statement. This statement should provide the following information, where applicable:

- Accession codes, unique identifiers, or web links for publicly available datasets
- A description of any restrictions on data availability
- For clinical datasets or third party data, please ensure that the statement adheres to our policy

The EM map and associated atomic model of the open-gate OCCM reported in this study have been deposited to the Electron Microscopy Data Bank (EMDB) and the RCSB Protein Data Bank (PDB) under accession numbers EMD-44441/PDB:9BCX, respectively. Other data that support the study are available from the

corresponding author upon reasonable request.
A source data file is available with the published article.

Research involving human participants, their data, or biological material

Policy information about studies with human participants or human data. See also policy information about sex, gender (identity/presentation), and sexual orientation and race, ethnicity and racism.

Reporting on sex and gender	N/A
Reporting on race, ethnicity, or other socially relevant groupings	N/A
Population characteristics	N/A
Recruitment	N/A
Ethics oversight	N/A

Note that full information on the approval of the study protocol must also be provided in the manuscript.

Field-specific reporting

Please select the one below that is the best fit for your research. If you are not sure, read the appropriate sections before making your selection.

☒ Life sciences ☐ Behavioural & social sciences ☐ Ecological, evolutionary & environmental sciences

For a reference copy of the document with all sections, see nature.com/documents/nr-reporting-summary-flat.pdf

Life sciences study design

All studies must disclose on these points even when the disclosure is negative.

Sample size	No statistical methods were used to predetermine sample size, as much data as possible was collected during allocated instrument session time. We collected 304,288 particles from 7500 micrographs for the original open-gate OCCM structure. For this study, we extracted 29,463 of those particles for the closed-gate OCCM.
Data exclusions	Data was excluded at different stages of image processing. Particles that did not meet the image resolution quality criteria or could not be averaged were excluded from the final structures, based on 2D averages and 3D map classification results. The method of data exclusion is common practice in the cryo-EM field to allow for the generation of interpretable 3D models.
Replication	Biochemical experiments were repeated at least two or three times as biological replicates.
Randomization	Experiments were not randomized due to the nature of the data not involving patients or animals. The experiments were not grouped and needed to be preformed in a particular order.
Blinding	Blinding is not applicable to this research, which does not entail randomization and does not involve patients or animals. The experiments required essential visual inspection of the data quality during data screening and data collection and so no blinding could be performed.

Reporting for specific materials, systems and methods

We require information from authors about some types of materials, experimental systems and methods used in many studies. Here, indicate whether each material, system or method listed is relevant to your study. If you are not sure if a list item applies to your research, read the appropriate section before selecting a response.

Materials & experimental systems

n/a	Involved in the study
☐	☒ Antibodies
☒	☐ Eukaryotic cell lines
☒	☐ Palaeontology and archaeology
☒	☐ Animals and other organisms
☒	☐ Clinical data
☒	☐ Dual use research of concern
☒	☐ Plants

Methods

n/a	Involved in the study
☒	☐ ChIP-seq
☒	☐ Flow cytometry
☒	☐ MRI-based neuroimaging

Antibodies

Antibodies used: Anti-Mcm5 mouse monoclonal antibody (1:5000). Gift from Bruce Stillman (CSHL).

Validation: The antibody has been described in the literature and referenced in the manuscript.

Plants

Seed stocks: N/A

Novel plant genotypes: N/A

Authentication: N/A
